# Single-cell characterisation of tissue homing CD4 + and CD8 + T cell clones in immune-mediated refractory arthritis

**DOI:** 10.1186/s10020-024-00802-1

**Published:** 2024-04-09

**Authors:** Dipabarna Bhattacharya, Jason Theodoropoulos, Katariina Nurmi, Timo Juutilainen, Kari K. Eklund, Riitta Koivuniemi, Tiina Kelkka, Satu Mustjoki, Tapio Lönnberg

**Affiliations:** 1 Hematology Research Unit Helsinki, University of Helsinki and Helsinki University Hospital Comprehensive Cancer Center, Helsinki, Finland; 2 Translational Immunology Research Program, University of Helsinki and Helsinki University Hospital, Helsinki, Finland; 3 iCAN Digital Precision Cancer Medicine Flagship, Helsinki, Finland; 4 Department of Computer Science, Aalto University, Espoo, Finland; 5 Faculty of Medicine, Clinicum, Translational Immunology Program, University of Helsinki, Helsinki, Finland; 6 ORTON Orthopaedic Hospital, Helsinki, Finland; 7 Department of Rheumatology, University of Helsinki and Helsinki University Hospital, Helsinki, Finland; 8 Department of Clinical Chemistry and Hematology, University of Helsinki, Helsinki, Finland; 9 Turku Bioscience Centre, University of Turku and Åbo Akademi University, Turku, Finland; 10 InFlames Flagship Center, University of Turku, Turku, Finland

**Keywords:** Arthritis, Autoimmunity, T cell, scRNA-seq, TCR-seq

## Abstract

**Background:**

Immune-mediated arthritis is a group of autoinflammatory diseases, where the patient’s own immune system attacks and destroys synovial joints. Sustained remission is not always achieved with available immunosuppressive treatments, warranting more detailed studies of T cell responses that perpetuate synovial inflammation in treatment-refractory patients.

**Methods:**

In this study, we investigated CD4 + and CD8 + T lymphocytes from the synovial tissue and peripheral blood of patients with treatment-resistant immune-mediated arthritis using paired single-cell RNA and TCR-sequencing. To gain insights into the trafficking of clonal families, we compared the phenotypes of clones with the exact same TCRß amino acid sequence between the two tissues.

**Results:**

Our results show that both CD4 + and CD8 + T cells display a more activated and inflamed phenotype in the synovial tissue compared to peripheral blood both at the population level and within individual T cell families. Furthermore, we found that both cell subtypes exhibited clonal expansion in the synovial tissue.

**Conclusions:**

Our findings suggest that the local environment in the synovium drives the proliferation of activated cytotoxic T cells, and both CD4 + and CD8 + T cells may contribute to tissue destruction and disease pathogenesis.

**Supplementary Information:**

The online version contains supplementary material available at 10.1186/s10020-024-00802-1.

## Background

Immune-mediated arthritis (IMA) is a group of diseases characterised by chronic inflammatory processes targeting primarily joints, but to some extent also other tissues. Rheumatoid arthritis (RA) is the most prevalent, and the most studied form of IMA, affecting ≤ 1% of the population worldwide, being more common in women than men and with a higher prevalence in developed than developing countries (Smolen et al. 2018; Finckh et al. 2022). RA is typically further divided into seropositive and seronegative arthritis diseases, depending on the presence of either rheumatoid factor or autoantibodies against citrullinated proteins (Ajeganova and Huizinga 2015; Willemze et al. 2012). Juvenile idiopathic arthritis (JIA) is the most prevalent form of IMA in children under the age of 16 (Martini et al. 2022; Prakken et al. 2011). JIA is a heterogeneous disease with several different subtypes, each characterised by distinct clinical and laboratory features. Similar to RA, also JIA can be divided into seropositive and seronegative subtypes, with the latter being more common (in 95% of patients (Hamooda et al. 2016)). While in most patients with IMA remission can be achieved with immunosuppressive medication (Singh 2022), curative treatments are lacking due to incomplete understanding of the disease aetiology.

The infiltration of lymphocytes, predominantly T cells, into synovial tissue is known to induce localised inflammation via the activation of macrophages and fibroblasts, thereby playing a significant pathogenic role in both RA and JIA (Prakken et al. 2011; Duke et al. 1982; Cope 2008). The synovium is enriched in memory T cells in RA (Thomas et al. 1992). They are instrumental in the propagation of autoantibodies and genome-wide association studies have indicated a strong link with the HLA-DR locus (Drongelen and Holoshitz 2017; Gregersen et al. 1987). Although CD4 + T cells are recognized as key drivers of RA pathogenesis (Weyand et al. 2000), CD8 + T cells with an inflammatory phenotype and oligoclonal T cell receptor (TCR) repertoire are also present in enriched numbers in synovial tissue (Cho et al. 2012; Carvalheiro et al. 2015; Savola et al. 2017), highlighting their potential role in the development and progression of RA. In a recent study (Moon et al. 2023), *GZMB* + *CD8* + T cells were shown to be activated by citrullinated autoantigens presented by MHC class I. Thus, identification of disease-promoting T cell populations and pathways that regulate their development, could provide targets for specific immunomodulation, while leaving the regulatory and protective populations intact.

Recent studies utilising TCRß deep sequencing and single-cell RNA sequencing (scRNA-seq) have provided a better understanding of synovial fluid and synovial tissue lymphocytes exhibiting significant phenotypic heterogeneity in patients with RA and IMA (Wu et al. 2021; Zhang et al. 2019; Stephenson et al. 2018; Henderson et al. 2016). For example, previous studies have revealed a novel synovial tissue T cell population expressing CXCL13, named as "peripheral helpers" (Tph). These cells resemble follicular T cells phenotypically and functionally, and are associated with active disease (Rao et al. 2017). Importantly, elevated levels of plasma CXCL13 have also been shown to correlate with disease activity, and T cells appear to be the main producers of CXCL13 in the synovial tissue in patients with seropositive RA (Stephenson et al. 2018; Greisen et al. 2014), supporting the pathologic relevance of the Tph subset. Traditionally, regulatory T cells (Tregs) control immune responses by suppressing effector T cells. Studies have reported a change in both number and function of Tregs in RA leading to breakdown of self-tolerance and autoimmune inflammation (Jiang et al. 2021; Rossetti et al. 2017).

While the homing of T cells to inflamed tissues is well-established in IMA, it is not known whether they are recruited from lymph nodes/blood or whether they differentiate and proliferate in synovial tissue. Ultimately, determining whether the presence of these cells represents stochastic and bystander activation or recruitment via TCR specificity may provide critical insights into the pathogenesis of RA.

In this study, we profiled paired synovial tissue and peripheral blood samples from three treatment refractory patients with IMA (one seropositive RA, one seronegative RA and one seronegative JIA) with paired single-cell RNA and TCR sequencing. Our results show that expanded T cell clones are enriched in synovial tissue samples, and overall, T cells in the synovial tissue have more inflamed phenotype. As a proportion of CD4 + and CD8 + T cell clones were shared between synovial tissue and circulation, we were able to validate the inflamed phenotype of T cells in the synovial tissue at the level of individual T cell clones.

## Methods

### Patients and samples

Synovial tissue samples from patients with immune mediated arthritis were collected during surgical synovectomy. All obtained tissue was removed based on clinical evaluation and no extra tissue was removed for the purpose of this study. Peripheral blood samples were collected using EDTA sampling tubes according to routine procedures. Written informed consent was received from all patients. This study was approved by the Helsinki University Hospital Ethics Committee (HUS/2989/2017).

### Isolation of peripheral blood mononuclear cells

Peripheral blood cells were pelleted by centrifugation (300 × *g*, 10 min) and EDTA plasma was collected and stored separately. The blood was first reconstituted to its original volume and then further diluted to 1.5 × volume with Phosphate-buffered saline with EDTA (PBS-E). The mononuclear cell (MNC) fraction was separated by density gradient centrifugation (800 × *g*, 25 min, no brake), using Ficoll-Paque PLUS (GE Healthcare). The collected MNCs were washed twice with PBS-E and cryopreserved in aliquots of approximately 5–10 × 10^6^ cells in FBS with 10% dimethyl sulfoxide (DMSO).

### Cell isolation from surgically removed synovial tissues

The tissues collected during synovectomy were cut into 2–4 mm pieces. The tissues were digested with the following enzyme mix: 4.7 ml Roswell Park Memorial Institute (RPMI) 1640 medium, 200 μl Enzyme H, 100 μl Enzyme R, and 25 μl Enzyme A (Miltenyi, Tumor Dissociation Kit, human, PN 130–095-929). The tissues were incubated with the enzyme mix for 2 × 30 min at + 37 °C in gentleMACS C tubes under rotation using the gentleMACS dissociator (Miltenyi). After digestion the samples were passed through a 70 μm MACS SmartStrainer (Miltenyi) and the strainers were washed with 20 ml RPMI 1640. The cells were pelleted by centrifugation (300 × *g*, 7 min) and the supernatants were removed. The cells were finally resuspended in 10 ml RPMI 1640. The cells were cryopreserved in aliquots of approximately 5 × 10^6^ cells in 10% DMSO in FBS.

### Flow cytometry

From synovial tissue samples, 5 × 10^5^ cells were stained with multitest CD3 FITC/CD8 PE/CD45 PerCP/CD4 APC reagent (BD, PN 342417) and anti-CD14- PacificBlue (Invitrogen, PN MHCD1428) for 10 min at RT in the dark. The cells were washed with 3 ml PBS-E and finally resuspended in 200 μl PBS-E. The samples were either measured the same day or resuspended in 200 μl fixation buffer (3 ml PBS-E + 37,5 μl formaldehyde). The samples were analysed using a FACSVerse instrument (BD).

### Fluorescence-activated cell sorting

The cells were suspended in staining buffer (1% BSA in PBS) and incubated with 2.5 μg per 100 μl Human Fc Block (BD Pharmingen, PN 564219) for 10 min at RT. The cells were pelleted by centrifugation and the supernatant was removed. The cells were incubated with antibodies for 30 min on ice in the dark. The PB and ST samples for sorting CD4 + and CD8 + T cells were stained with the following antibodies: CD8(RPA-T8)-BV421 (BD, PN 562428), TCR(WT31)-FITC (eBioscience, PN 11-9955-42), PD1(EH12.1)-PE (BD, PN 560795), CXCR5(J252D4)-APC (BioLegend, PN 356907), CD4-PerCp-Cy5.5 (BD, PN 332772), and CD45RA(HI100)-APC-Cy7 (BioLegend, PN 304127). The ST samples for sorting αβ T cells were stained with CD3(UCHT1)-APC (BioLegend, PN 300411) and TCRα/β(IP26)-PE (BioLegend, PN 306707). The PB samples for sorting of CD45 + leukocytes were stained with CD45(2D1)-APC-H7 (BD, PN 560178). After staining the samples were washed once and resuspended in staining buffer. Sorting was performed with a Sony SH800 instrument using a 100 μm nozzle.

### Statistical analysis

P-values were calculated with Fisher’s exact test and corrected with Benjamini–Hochberg adjustment. All calculations were done with R (version 4.2.1).

### Single cell capture and preparation of sequencing libraries

Prior to single-cell capture, the cells were suspended in 0.04% BSA in PBS in concentrations varying from 100 to 1300 cells per μl. Single-cell capture and preparation of single-cell RNA-seq and TCRαβ libraries was performed using Chromium Single Cell 5′ Library & Gel Bead Kit V1, Chromium Single Cell Human T Cell V(D)J Enrichment Kit, and the Chromium Controller instrument (10x Genomics), following manufacturer’s instructions (document CG000086). The PCR steps were carried out with a Veriti cycler (Applied Biosystems / Thermo Fisher), using 14 cycles for the amplification of the full-length cDNA, 10 cycles for the TCR target enrichment PCR reactions, 14 cycles for the sample index PCR of the gene expression libraries, and 9 cycles for the TCR libraries.

### Sequencing

The libraries were sequenced using an Illumina HiSeq 2500 or an Illumina NovaSeq 6000. For gene expression libraries the following read length configuration was used: Read1 = 26, i7 = 8, i5 = 0, Read2 = 91. The TCR-enriched libraries were sequenced either using the same configuration or with: Read1 = 150, i7 = 8, i5 = 0, Read2 = 150.

### Data processing and quality control

All samples were filtered using the following criteria. Low quality cells, as determined by the fulfilment of conditions stated below were removed from the analysis: > 15% mitochondrial transcripts, < 10% or > 50% ribosomal transcripts, < 250 or > 4500 expressed genes or < 1000 or > 20,000 UMI counts. All publicly available datasets were sampled similarly to remove low quality cells. Single cell analysis was done with the Seurat tool (Satija et al. 2015). Samples were merged with scVI (Lopez et al. 2018) with default parameters to reduce batch effect and each sample was used as a batch. The latent embeddings obtained from scVI were then further used for graph-based clustering and uniform manifold approximation and projection (UMAP) dimensionality reduction as implemented in Seurat (4.3.0). The datasets were scaled with 3,000 most highly variable genes with the FindVariableFeatures-function. Proliferation score was defined by the expression of the genes *LIF, IL2, CENPV, NME1, FABP5, ORC6, G0S2, GCK* and calculated by the AddModuleScore-function as defined by Tirosh et al. (Tirosh et al. 2016). Cell cycle scoring and prediction was performed using the CellCycleScoring – function as implemented in the Seurat toolkit. Integrating of TCR data to the transcriptome was done using the screpertoire (Borcherding et al. 2020) tool as implemented in R. Enrichment analyses were performed with upregulated DE genes (p < 0.05, |Log2FC| > 0.5) with ClusterProfiler (Yu et al. 2012) using hypergeometric testing. GLIPH2 (Sharma et al. 2021) (v 1.0.0) with default parameters was run on a remote server to group TCRs into amino acid level similarity groups. All patterns identify as “single” in GLIPH2 were removed from further downstream analysis to reduce noise. TCRβ-sequencing data from Henderson et al. (Henderson et al. 2016) was downloaded from ImmunoSEQ ANALYZER and filtered to remove all TCRs with non-functional clonotypes as low-quality reads.

## Results

### Synovial tissue and peripheral blood represent transcriptionally different niches and clonally expanded T lymphocytes are enriched in the synovial tissue

**Fig. 1:**
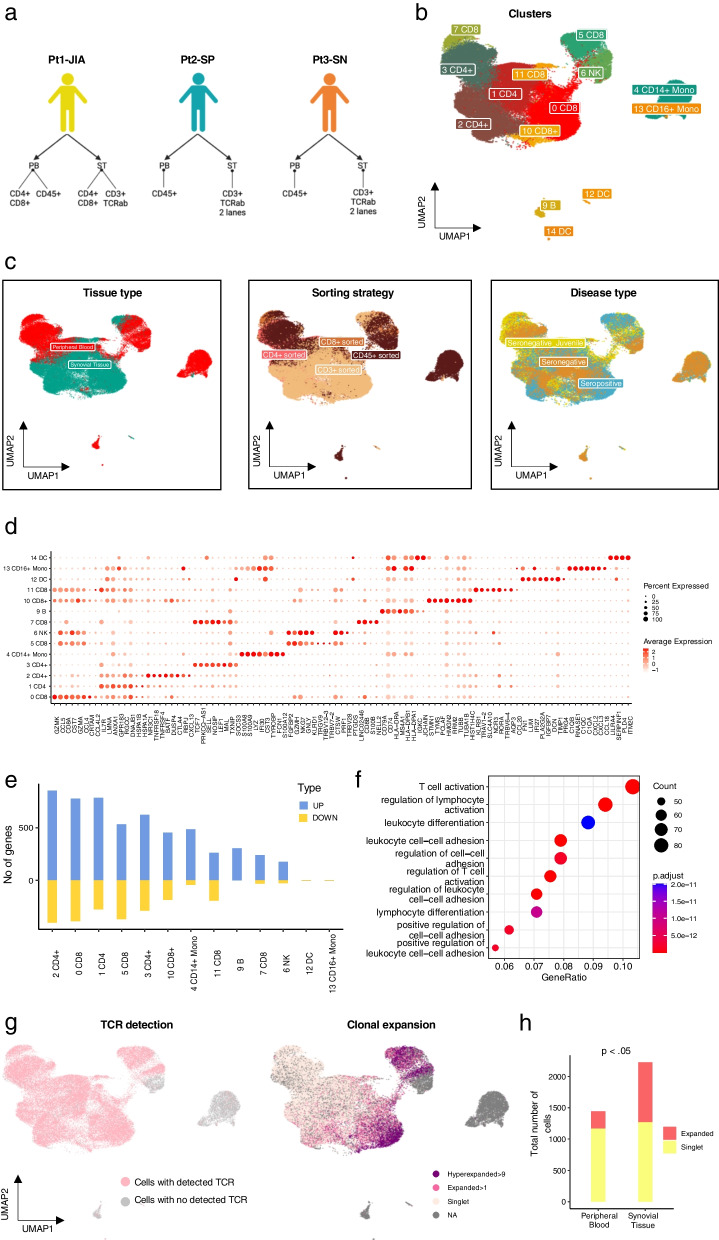
Phenotypic characterization of CD45 + cells in the cohort.** a** Overview of the study—Patient cohort and study samples.** b** Uniform Manifold Approximation and Projection (UMAP) representation and unsupervised clustering of all cells from the IMA samples (n = 3) profiled with scRNA + TCRαβ-seq.** c** Left: UMAP representation of the cells from ST and PB in each cluster as identified in our study. Middle: UMAP representation of the cells as per sorting strategy in each cluster as identified in our study. Right: UMAP representation of the cells representing different disease types.** d** Dot plot with scaled expression of top 8 DE genes between IMA phenotype clusters (Padj < 0.05, calculated as Bonferroni corrected Wilcoxon test).** e** Number of DE genes both upregulated and downregulated between cells from ST and PB for each cluster (Padj < 0.05, |Log2FC| > 0.5 calculated with Bonferroni corrected two-sided t-test).** f** Pathway enrichment analysis with GO biological processes (Hypergeometric testing).** g** Left: Focused UMAP of all cells with TCR from panel 1b. Right: Proportion of the cells identified as Hyperexpanded (> 9), Expanded (> 1) and Singlet as identified in the UMAP.** h** Cells detected as expanded versus cells detected as singlets in ST and PB in our data shows that expanded cells are enriched in ST (Padj < 0.05, Benjamini–Hochberg corrected Fisher’s one-sided exact test)

To characterise the phenotypic and clonal diversity of T cells in IMA, we analysed paired synovial tissue (ST) and peripheral blood (PB) samples from three patients. The patients had been diagnosed with seronegative juvenile arthritis (Pt1-JIA), seropositive RA (Pt2-SP) and seronegative RA (Pt3-SN), and at the time of sampling they were refractory to treatment (Fig. 1a, Additional file 2: Table). All patients have had prior treatments including anti-TNF, anti-CTLA4 and JAK inhibitors. Available clinical information is presented in the Additional file 2: Table.

We profiled flow sorted T cells and CD45 + leukocytes from ST and PB using 10x Genomics 5´-scRNA + V(D)J-seq (Additional file 1: Fig. S1a). In addition, in one set of samples (from Pt1-JIA), CD4 + and CD8 + T cells were sorted and processed separately to corroborate the scRNA-seq based annotation of these cell types (Additional file 1: Fig. S1a). Altogether, we captured 68,610 cells passing quality control thresholds (Additional file 1: Fig. S1b). Batch effects between individual samples were corrected for using scVI (Lopez et al. 2018) (Additional file 1: Fig. S1c, d and Methods). We integrated the data from all individual samples and visualised the subpopulation structure using UMAP dimensionality reduction as implemented in the Seurat package (Satija et al. 2015) (Fig. 1b, Additional file 1: Fig. S2a). Clusters were primarily identified based on the expression of canonical markers (Additional file 1: Fig. S2b). We observed strong transcriptomic differences between the tissue of origin in concordance with previous reports (Wu et al. 2021; Argyriou et al. 2022) (Fig. 1c, leftmost panel). Although our patient cohort represented different disease subtypes and sorting strategies, the subpopulation structures did not exhibit marked differences based on disease type or patient (Fig. 1c, middle—right panel, Additional file 1: Fig. S2c–e). The top 8 differentially expressed (DE) genes from each of the clusters are represented in Fig. 1d. To better identify sources of heterogeneity in our clusters we computed differentially expressed (DE) genes between all cells from ST and all cells from PB for each cluster and found that the highest number of DE genes were found in CD4 + and CD8 + T cell clusters (Fig. 1e). As expected, cells from the synovial tissue represented a more inflamed (*LMNA *+ *, CREM* +) and activated (*CXCL13*+ *, CCL5* + *CCL4* + *,* and *RGCC+)* phenotype (Additional file 1: Fig. S3a). Pathway enrichment analysis using GO biological processes as the reference gene set identified that cells in ST are enriched for processes including T cell activation and leukocyte adhesion (Fig. 1f). We additionally compared the compound expression values of gene modules from Azizi et al. (2018) and Long et al. (2015) between ST and PB, further confirming a more activated, inflammatory and exhausted state among the synovial cells (Additional file 1: Fig. S3b). Notably, the expression of each module was significantly higher among clonally expanded cells (Additional file 1: Fig. S3c).

To better understand how clonally expanded cells are distributed between PB and ST, we then integrated the single-cell TCR data from both tissue types. Cells with detected TCRs (either α or β or both chains) are highlighted in Fig. 1g. Clones were defined as either expanded (at least two cells with identical CDR3b) or singlets (unique CDR3b in each cell). Additionally, clones were categorised as hyperexpanded (at least ten cells with identical CDR3b) Fig. 1g right panel). As reported before (Seder and Ahmed 2003), CD8 + T cells had a higher number of hyperexpanded clones as compared to CD4 + T cells. We measured the overall clonality in all CD45 sorted samples (3115 cells from ST and 2473 from PB) using only one sequencing library per patient (to ensure that there was no sequencing depth related bias for samples that were profiled twice). Overall, ST T cells were found to be less clonal (Gini index 0.358) as compared to PB (Gini index 0.584) as the clones in PB were substantially bigger (Additional file 1: Fig. S3d, e). However, by comparing the number of expanded clones versus singlets, we found that ST compartment was enriched for expanded cells (Fig. 1h) (Fisher's exact test, p value < 2.2e-16, odds ratio 0.3).

### Cytotoxic, inflamed, and highly activated CD4 + T cells with proliferation-associated gene expression signatures dominate the synovial tissue samples

**Fig. 2:**
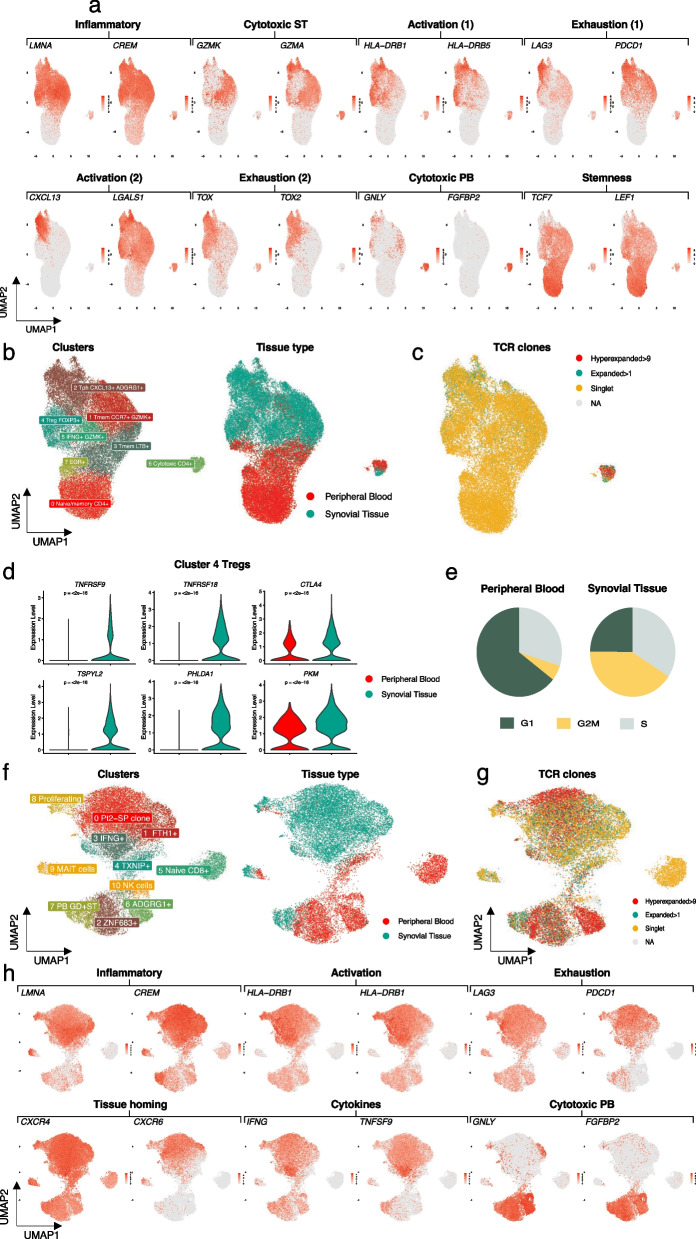
CD4 + and CD8 + T cells and their phenotypes in IMA.** a** Feature plot of top DE genes between ST and PB CD4 + T cells (Padj < 0.05, |Log2FC| > 0.5 calculated with Bonferroni corrected two-sided t-test).** b** Left: UMAP representation of the re-clustered CD4 + T cells. Right: UMAP representation of the tissue origin.** c** UMAP representation of TCR clones identified as Hyperexpanded (> 9), Expanded (> 1) and Singlet.** d** Violin plot of top DE genes between ST and PB in re-clustered Tregs (Padj < 0.05,  |Log2FC| > 0.5 calculated with Bonferroni corrected two-sided t-test).** e** Proportion of cells in different phases of cell cycle in all CD4 + cells in PB and ST.** f** Left: UMAP representation of the re-clustered CD8 + T cells. Right: UMAP representation of the tissue origin.** g** UMAP representation of TCR clones identified as Hyperexpanded (> 9), Expanded (> 1) and Singlet.** h** Feature plot of top DE genes that are different between ST and PB in CD8 + T cells (Padj < 0.05, |Log2FC| > 0.5 calculated with Bonferroni corrected two-sided t-test)

To further narrow down on the differences between the tissue types, we next subsetted the CD4 + and CD8 + T cells and analysed them separately. DE analysis of the CD4 + T cells from ST (17,720 cells) and PB (16,414 cells) confirmed that the ST cells represented a more inflamed (*LMNA* + *CREM* +), activated (*CXCL13* + *LGALS1* +), and exhausted (*LAG3* + *PDCD1* +*)* phenotype (Fig. 2a-b). Additionally, the ST cells uniformly expressed higher amounts of transcription factor *TOX* (Fig. 2a)*,* a known marker for repeated antigen stimulation (Maschmeyer et al. 2021) and Th1 subset differentiation (Page et al. 2018; Guo et al. 2020). In contrast, PB cells had uniformly higher expression of *TCF7* (Fig. 2a-b)*,* a marker for T cell stemness and self-renewal capacity (Escobar et al. 2020) that has also been proposed to have a significant role in activating B cells via T_fh_ (Xu et al. 2015; Wu et al. 2015).

Subclustering of the CD4 + T cells resulted in 8 clusters (Fig. 2b left panel), with very limited overlap between the two tissues (Fig. 2b right panel, Additional file 1: Fig. S4a–c). Clusters 0 and 7 consisted primarily of PB cells. The cells in cluster 0 expressed genes associated with naïve phenotype such as *TCF7*, *SELL*, and *CCR7* (Additional file 1: Fig. S4d). The cells in cluster 7 expressed immediate early response genes (Neeb et al. 2012) *EGR1*, *IER2*, *JUN*, and *FOS* (Additional file 1: Fig. S4d), resembling a population of *EGR1* + naive CD4 + T cells described in a recent report by Argyriou et al. (Argyriou et al. 2022). Clusters 3, 4, and 6 consisted of cells from both tissues. The cells in cluster 3 expressed memory cell-associated genes *IL7R* and *LTB*. Cluster 4 consisted of regulatory T cells expressing *FOXP3*, *IL2RA* (encoding CD25), *RTKN2*, and *IKZF2* (encoding Helios). The cells in cluster 6 expressed high levels of genes associated with cytotoxic phenotype, including *FGFBP2*, *GNLY*, and *NKG7* (Additional file 1: Fig. S4d). Clusters 1, 2, and 5 were enriched to the ST. Cluster 1 consisted of memory cells co-expressing *GZMK* and *IL7R*. Cluster 2 consisted of ST cells expressing *CXCL13*, *ADGRG1*, *PDCD1* (Additional file 1: Fig. S4d), and other markers associated with a peripheral helper phenotype (Argyriou et al. 2022). In addition, these cells expressed several known cell division markers (*GOS2*). The cells in cluster 5 expressed elevated levels of *IFNG* and *TNF* together with *GZMK* and stress-associated transcripts *HSPA6* and *DNAJB1* (Additional file 1: Fig. S4d). By comparing the TCR data with the clustering, we found that expanded clones were present in cluster 2 (Tph) and most significantly in cluster 6 (cytotoxic) (Fig. 2c). Notably, the clonally expanded cluster 6 was shared between Pt1-JIA and Pt2-SP (Additional file 1: Fig. S4c).

The cluster of Treg cells (cluster 4) was identified based on the expression of the canonical *FOXP3* gene (Additional file 1: Fig. S4e). Interestingly, while falling into the same cluster, the Tregs from blood were phenotypically different from the ones in ST, with the ST Tregs comprising a more activated phenotype (Fig. 2d, Additional file 1: Fig. S4d). DE gene analysis showed that Tregs from ST expressed *TNFRSF9*, a marker for T cell activation (Bartkowiak and Curran 2015) and co-inhibitory receptor *CTLA4* (Fig. 2d). The DE genes also included inhibitor of cell cycle *TSPYL2*, regulator of TLR4 signalling pathway *PHLDA1*, and *PKM* (Peng 2022; Xu et al. 2019) (Fig. 2d, Additional file 1: Fig. S4e). The TCR data indicated modest clonal expansion within the Treg cluster: 17% of the Tregs in ST were expanded as compared to 2% in PB (p < 0.05, Fisher’s exact test).

To gain further insights into the developmental relatedness of the observed clusters, we assessed the extent of clonal overlap amongst them using Morisita’s index (Rempala and Seweryn 2013). While we found that the overall frequency of shared TCR clones was low, a higher index score (translating to higher similarity) was found between clusters 1 (Tmem *GZMK* + *CCR7* +) and 6 (cytotoxic CD4 +) (Additional file 1: Fig. S4f). The clonality between ST (Gini index 0.21) and PB (Gini 0.203) was comparable but the expanded clones, were still enriched to ST (p-value < 2.2e-16, Fisher's exact test).

Interestingly, cells from ST preferentially belonged to the G2/M phases (both overall and in all individual clusters) whereas in PB cells were mostly in G1 phase (Fig. 2e, Additional file 1: Fig. S4g). Additionally, the expression of proliferation-associated genes *FABP5, G0S2,* and *MKI67* was significantly higher in ST than in PB (Additional file 1: Fig. S4h). Cluster 2 was enriched in cells assigned to G2/M phases, expressed high levels of proliferation-associated genes, and exhibited clonal expansion, suggesting antigen-driven proliferation in the ST. As the ST samples were mechanically dissociated, we cannot rule out stress-related response, but interestingly, this effect was stronger in expanded cells than in singlets, suggesting that the observed differences are related to clonal expansion (Additional file 1: Fig. S5a). We also performed additional tests to check if tissue related differences were technical artifacts. scVI was used with tissue of origin as batch key to mitigate possible batch effects but this did not remove the signature as cells from PB and ST still formed their distinct clusters (Additional file 1: Fig. S5b). To further validate the finding, we also analysed a single-cell RNA-seq dataset from both PB and synovial membrane of 20 patients with either seropositive or seronegative RA recently published by Wu et al. (Wu et al. 2021) (Methods). In concordance with our data, there was an overall higher proliferation score (defined by combined expression of *LIF, IL2, CENPV, NME1, FABP5, ORC6, G0S2,* and* GCK*) among the synovial membrane derived CD4 + T cells (Additional file 1: Fig. S5c, d).

### Clonally expanded CD8 + T cells are predominantly localised in the synovial tissue in patients with seronegative disease

We next analysed CD8 + T cells and subclustered them separately to achieve a higher resolution of the phenotypes and their relationship with the TCR clonotypes. The clusters were annotated based on the top DE genes (Additional file 2: Table) and known CD8 + T cell markers. As with the CD4 + T cells, we observed high heterogeneity between the CD8 + T cells from ST and PB (Fig. 2f, Additional file 1: Fig. S6a–c). However, the CD8 + T cells represented a more heterogeneous population as the top expanded clone(s) from each patient occupied a distinct space in the UMAP representation of clusters. Both tissue types (PB and ST) harboured a high number of expanded and hyper expanded clones (Fig. 2g).

Sub-clustering of the CD8 + T cells resulted in 9 clusters (Fig. 2f). By comparing the top DE genes between ST and PB we found that similar to the CD4 + T cells, the CD8 + T cells in ST represented an inflamed (*CREM, LMNA*), activated (*HLA* genes, *LAG3*) and less cytotoxic (*GNLY, FGFBP2*) effector memory phenotype (*GZMK*) (Fig. 2h)*.* However, CD8 + T cells lacked expression of the chemokine *CXCL13* and the galectin *LGALS1.* Similar to the CD4 + T cells, ST CD8 + T cells were enriched in the G2/M phases of cell cycle (Additional file 1: Fig. S6d).

Clusters 2, 5, 6, and 10 consisted primarily of PB cells (Fig. 2f right panel). The cells in clusters 2 and 6 expressed effector-related genes *FGFBP2*, *GNLY*, and *GZMH* (Fig. 2h, Additional file 1: Fig. S6e). In addition, cluster 2 cells expressed *ZNF683* (encoding Hobit (Mackay et al. 2016)) and cluster 6 cells expressed cytotoxicity-associated genes *KLRC1* and *KLRC2* (Additional file 1: Fig. S6e). Cluster 5 consisted of cells expressing genes associated with a naive phenotype, including *LEF1*, *TCF7*, and *SELL* (Additional file 1: Fig. S6e). Clusters 4, 7, 8, and 9 consisted of cells from both tissues. The top upregulated marker gene for cluster 4 was *TXNIP*, a key regulator of oxidative stress and inflammatory response (Jiang et al. 2022). Recently, *TXNIP* has been suggested to promote M1 pro-inflammatory polarisation thereby inhibiting the M2 anti-inflammatory polarisation in RA (Li et al. 2021). Cluster 7 represented a highly clonal cluster and consisted of top clones from multiple patients. Cluster 8 was identified as highly proliferative along with expression of cell cycle genes (*MKI67, STMN1*) (Additional file 1: Fig. S6e).

Clusters 0, 1, and 3 were enriched for ST cells. Cluster 0 harboured the hyper expanded clone from Pt2-SP and top markers included HLA genes *(HLA-DRB5*, *HLA-DRB1*, *HLA-DQA1*, *HLA-DRA*) and chemokine *CXCR6* known to have a role in B cell migration in RA (Zhao 2022) (Fig. 2h). The top upregulated genes for cluster 1 included *FTH1*, previously shown to be upregulated in the synovium of RA patients (Ling et al. 2022). Both clusters 0 and 1 upregulated expression of inflammatory markers *RGCC* and *CREM*. Cluster 3 represented a highly activated signature with top upregulated genes including inflammatory cytokines (*IFNG, TNF, TNFSF9*) and transcription factors (*JUN, FOSB*) (Fig. 2h, Additional file 1: Fig. S6e).

### Cells within clonal families acquire a more proliferative, inflammatory, and activated phenotype when trafficking from PB to ST

Lastly, we examined the overlap of TCR sequences between the ST and PB in each individual patient. We pooled all CD4 + and CD8 + T cells separately (Methods) and further grouped all cells with identical CDR3b aa sequences into unique clones. Since there were no shared expanded clones between the patients, the results are reported at individual patient level.

**Fig. 3:**
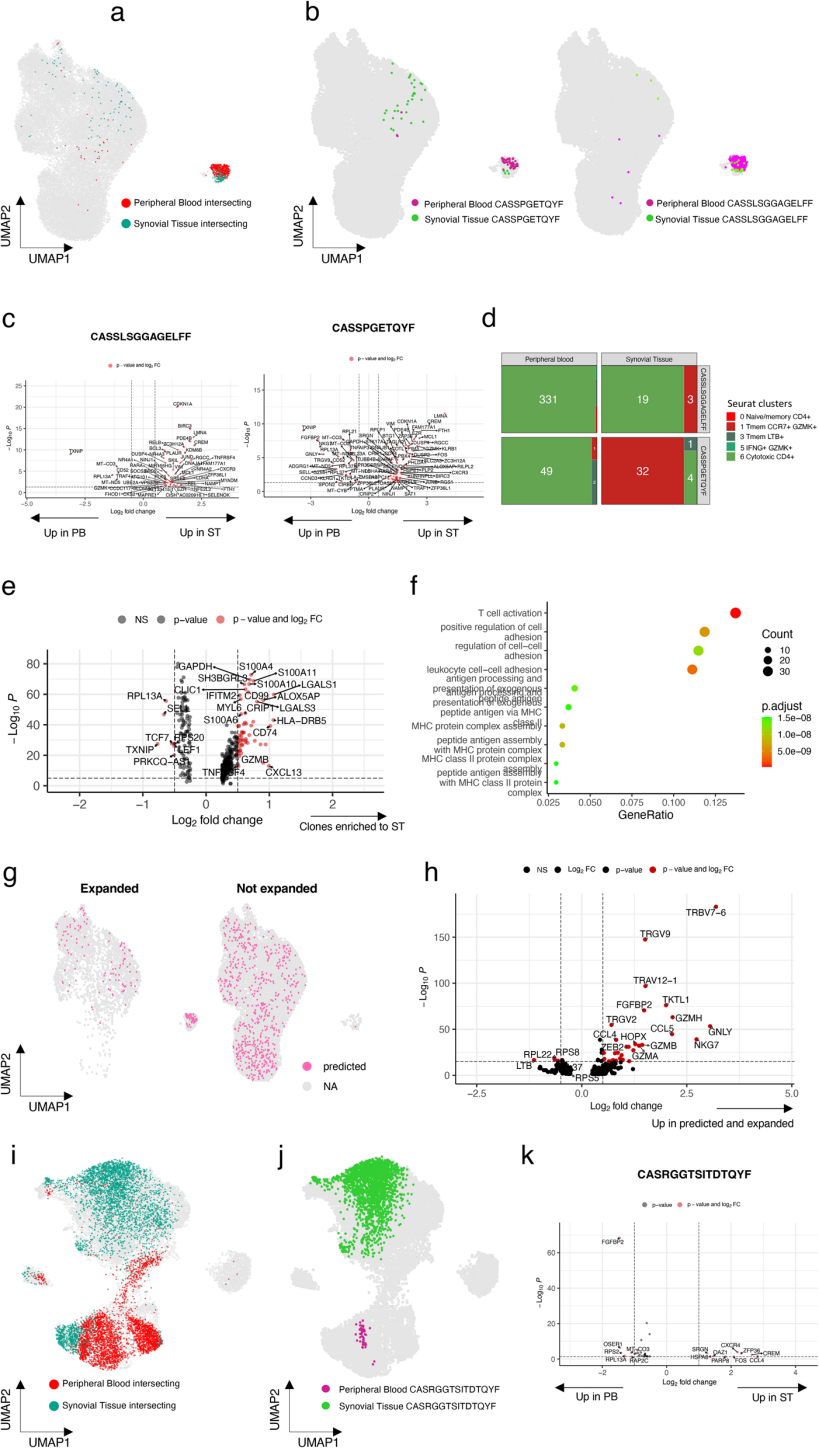
Clonal trafficking of CD4 + and CD8 + T cells between synovial tissue and peripheral blood.** a** All common clones (defined by the exact same CDR3b sequence) in CD4 + cells between ST and PB presented in the UMAP representation from panel 2b.** b** Cells with clones CASSPGETQYF (left panel) and CASSLSGGAGELFF (right panel) as projected in UMAP representation from 2b.** c** Left: DE genes between ST and PB cells sharing the same CDR3b sequence CASSPGETQYF in Pt1-JIA (Padj < 0.05, |Log2FC| > 0.5 calculated with Bonferroni corrected Wilcoxon test). Right: DE genes between ST and PB cells sharing the same CDR3b sequence. CASSLSGGAGELFF in Pt1-JIA (Padj < 0.05, |Log2FC| > 0.5 calculated with Bonferroni corrected two-sided t-test). **d** Number of cells with CDR3b clones CASSPGETQYF and CASSLSGGAGELFF in different clusters as identified in UMAP representation from 2b. **e** DE genes of all CD4 + T cells that are enriched in ST as compared to PB (Padj < 0.05,  |Log2FC| > 0.5 calculated with Bonferroni corrected two-sided t-test). **f** Top upregulated GO-BP pathways in CD4 + T cells that are enriched in ST as compared to PB (Padj < 0.05, Benjamini–Hochberg corrected Fisher’s one-sided exact test on differentially expressed genes). **g** UMAP representation of all CD4 + T cells predicted to have a motif in GLIPH2 analysis as seen in expanded versus non expanded cells. **h** DE genes of CD4 + T cells that are predicted to have a pattern in GLIPH2 and are enriched to ST as compared to PB. **i** All common clones (defined by the exact same CDR3b sequence) in CD8 + T cells between ST and PB presented in the UMAP representation from panel 2f. **j** Cells with clones CASRGGTSITDTQYF as projected in UMAP representation from 2f. **k** DE genes between ST and PB cells sharing the same CDR3b sequence CASRGGTSITDTQYF in Pt2-SP (Padj < 0.05, calculated with Bonferroni corrected two-sided t-test)

In the CD4 + compartment, we searched for all TCRs that were found in both PB and ST and that were detected in at least three cells in both tissues. We found altogether 12 such intersecting TCR clones pertaining to 637 cells, and they were derived from two individual patients, Pt1-JIA and Pt2-SP (Fig. 3a, Additional file 1: Fig. S7a–c, Additional file 1: Table). Majority of these clones were coming from PB (458 cells from PB and 179 from ST) and were hyperexpanded in PB as compared to ST (Additional file 1: Fig. S7d) and thus could potentially reflect immune responses to previous infections. However, we found no matches for common viral or bacterial epitopes in the existing VDJ database (Goncharov et al. 2022) for any of the intersecting clones. All discovered TCR matches were only for singlets (Additional file 1: Fig. S7e).

We compared the phenotype of the clones between PB and ST, and only two clones (CDR3b sequence—CASSPGETQYF and CASSLSGGAGELFF), both originating from the PT1-JIA had significant DE genes between the two tissue types (Fig. 3b left and right panel). Both clones were enriched to PB and to cytotoxic CD4 + cluster 6 (p < 0.05, Fisher’s exact test). Top DE genes from both clones included the genes *LMNA, RGCC, CREM, CDKN1A,* and *GPR35* (Fig. 3c) in the ST as compared to PB, suggesting that also cells belonging to bystander clones can develop an inflammatory ST phenotype when trafficked to the ST. Both clones were mainly detected within the cytotoxic cluster 6 (Fig. 3d). We found only one clone (CASSAFSAGATNEQFF) enriched to the ST but due to low cell numbers could not find any DE genes between the tissues for this clone.

Since this approach was somewhat limited by the number of cells, we then pooled all T cell clones that were enriched in the ST and found 113 clones (p < 0.05, Fisher's exact test). DE gene analysis showed that ST enriched clones have a more activated profile (Fig. 3e).

Pathway enrichment analysis with hypergeometric testing against the GO Biological Process database identified T cell activation and positive regulation of cell adhesion as the top hits (Fig. 3f). Using GLIPH2 (Sharma et al. 2021), we used publicly available data from a cohort of JIA patients, as reported by Henderson et al. (Henderson et al. 2016), to check TCR level similarity between the two cohorts. All patterns found in the healthy samples and Lyme disease patients were removed to retain possible RA associated motifs only. We found common motifs (Additional file 2: Table) between these two cohorts in some of the hyperexpanded clones from PB and ST (Fig. 3g) although with disparate phenotypes (Additional file 1: Fig. S7f). Interestingly, TCRs which were predicted to target similar antigens, were enriched to both expanded and intersecting clones. Additionally, by performing DE gene analysis we found that predicted TCRs with similarity were more cytotoxic (*GZMH, GNLY, NKG7, GZMB, GZMA*) and they overexpressed the TRBV7-6 gene (Fig. 3h). Previous publications have shown that TRBV7 family is overexpressed in JIA (Henderson et al. 2016).

In CD8 + T cells, as the average clone size was much bigger, a significantly higher number of clones (n = 101, pertaining to 7409 cells) was shared between PB and ST and in between clusters (Fig. 3i, Additional file 2: Table, Additional file 1: Fig. S7g, h). 8 of these clones were enriched to the ST as compared to PB. Interestingly, Pt2-SP harboured a large clone in the ST consisting of 27% of the CD8 + T cells (CDR3b sequence – CASRGGTSITDTQYF). This clone was also detected in PB with a much smaller frequency of only 2% and in a phenotypically different cluster, indicating local proliferation of specific T cell clones in the synovium (Fig. 3j, Additional file 1: Fig. S7g, h). By performing a DE gene analysis between cells with the same TCR but between different tissues of origin, we found that the top upregulated genes in ST were *CCL4* and *CREM* (Fig. 3k).

## Discussion

Recent studies in RA have utilised single-cell technologies to elucidate the mechanisms underlying clonal proliferation in inflamed joints, with a particular emphasis on CD4 + T cells. These studies have enabled higher resolution and the identification of novel subcategories of disease-promoting T cells. For instance, recent reports have identified two subcategories of CD4 + Tph cells distinguished by differential expression of *CXCL13*, with GPR56 as the delineating marker (Argyriou et al. 2022). Our results from paired PB and ST samples from three patients with distinct subtypes of IMA confirm the existence of the Tph subpopulation in the ST and support a model in which this disease-associated tissue-infiltrating population develops and proliferates locally, driven by repeated encounters with yet-to-be characterised antigens.

Tregs are known to be dysregulated in RA. Using TCR sequencing, Rosetti et al. (Rossetti et al. 2017) reported a new subclass of synovial Tregs called inflammation-associated Treg which recirculate back into the bloodstream during active infection and share repertoire level similarity with pathogenic effector T cells. We similarly found that during active disease Tregs are more abundant in the ST than in blood and that they are phenotypically more activated (expressing *TNFRSF9 *and* CTLA4).* We also found that hyperexpanded T cell clones are shared between Tph and Tregs, which bolters the hypothesis that during active inflammation CD4 + T clones can transiently upregulate *FOXP3* (Pillai et al. 2007; Yadav et al. 2013).

Although one of the major limitations of our study is that our patient cohort consists of three patients having three different forms of treatment resistant inflammatory arthritis, our results show that CD4 + T cell clones across diseases represent a relatively homogeneously activated, inflamed and proliferative phenotype in the ST samples. In addition, as we combined single-cell TCR sequencing to transcriptome profiling, we were able to examine individual T cell clones trafficking between the two tissue types. Overall, SN RA and JIA samples harboured much smaller T cell clones and these clones were usually bigger in PB than in ST. In the SN sample, we found two CD4 + T cell intersecting clones between the tissue types, and DE gene analysis confirmed that when clones home to ST they express more inflammation and activation related genes and upregulate the TNFA signalling via NFKB pathway. In contrast, the SP sample represented a more distinct TCR repertoire and harboured a big CD8 + clone (27%) in the ST compartment. Since this clone was also found in PB at a much lower proportion (2%), we hypothesise that this clone has undergone local proliferation at the inflamed site. It would be of interest to discover the antigen target of this expanded clone, but at least based on the database searches it does not target any known viral antigen. A putative list of autoantigens already exists for seropositive RA, but the number of studies looking at TCR level similarities between these autoreactive TCRs is still deficient. Previously, citrullinated tenascin C reactive TCRs from RA patients were shown to share CDR3 motifs and the TRBV20-1 gene with HLA-DRB1*04:01 genotype (Sharma et al. 2021), but our clone did not match with these reported shared CDR3s.

Taken together, our findings indicate that the local milieu of cytokines and signals in the ST induces a pervasive activation and inflammation signature within the T cell compartment. Our data confirms that this phenotypic shift occurs within clonal families and is not caused by selective recruitment of activated cells. Future studies can hopefully address the disease-promoting cellular interactions in the ST in both spatial and temporal resolution.

## Conclusions

Our results indicate that the local environment in the synovium promotes activation and proliferation of cytotoxic T cells, resulting in tissue-resident clonally expanded inflammatory populations. Both CD4 + and CD8 + T cells are likely to contribute to tissue destruction and disease pathogenesis. Our results indicate that synovial peripheral helper cells are present in different disease subtypes and might present an attractive population for targeted interventions.

### Supplementary Information

**Additional file 1: Figure S1.** Phenotypic characterization of CD45 + cells in the cohort. **a** The patient cohort and the experimental design. Tissue types, cell sorting approaches, and sample processing batches have been highlighted. **b** Quality control of cells: analysis excluded cells that were considered low-quality based on the following criteria: > 15% reads from mitochondrially-encoded transcripts, < 10% or > 50% ribosomal transcripts, < 250 or > 4,500 expressed genes, or < 1,000 or > 20,000 UMI counts. **c** UMAP representation of all cells from the IMA samples (n = 3) profiled with scRNA + TCRαβ-seq without any batch correction. d) UMAP representation of the cells representing different tissue of origin in each cluster as identified in UMAP presented in 1c. **Figure S2.** Phenotypic characterization of CD45 + cells in the cohort (cont.). **a** UMAP representation of all CD45 + cells from IMA samples (n = 3) profiled with scRNA + TCRαβ-seq. Clusters were defined based on the expression of canonical markers and known cell surface expression of different CD45 + cell types. **b** The expression of canonical markers of different cell types in each cluster as identified in UMAP in 1b. **c** Proportion of cells from individual patients in each cluster. **d** Absolute number of cells from individual patients in each cluster. **e** UMAP representation of the IMA dataset split by individual patients. **Figure S3.** Phenotypic characterization of CD45 + cells in the cohort (cont.). **a** Feature plot of inflammation (*LMNA*, *CREM*) and activation (*CXCL13*, *CCL5*, *CCL4* and *RGCC*) associated genes identified in the UMAP 1b. **b** Activation, inflammation, exhaustion, and inhibitory module scores as compared between tissues. **c** Activation, inflammation, exhaustion, and inhibitory module scores as compared between cells belonging to expanded versus not expanded clones. **d** Clonality index (Gini, higher Gini denotes more clonal) between ST and PB. **e** Clonality index (Gini) between ST and PB in individual samples. **Figure S4. a** Phenotypic characterization of CD4 + T cells in the cohort. Proportion of the cells from ST and PB in each of the CD4 + T cell clusters shown in Fig. 2b. **b** Relative proportion of the cells from ST and PB in each cluster as identified in the UMAP in Fig. 2b, each cluster represents 100% of the cell population. **c** Left: Proportion of the cells from individual patients as identified in the UMAP in Fig. 2b. Right: Proportion of the cells from individual samples as identified in the UMAP in Fig. 2b. **d** Expression of phenotypic markers in CD4 + T cell clusters. **e** Expression of phenotypic markers as identified in the UMAP 2b. **f** Clonal overlap (as measured by TCR similarity by Morisita index) between clusters as identified in the UMAP 2b. **g** Proportion of cells in different phases of cell cycle in each cluster as identified in the UMAP 2b in all CD4 + T cells in ST and PB. **h** The expression of proliferation-associated transcripts *G0S2*, *FABP5*, and *MKI67* in ST compared to PB. (Bonferroni corrected two-sided t-test). **Figure S5. a** Phenotypic characterization of CD4 + cells in the cohort (cont.). Proliferation score (based on the expression of proliferation associated genes) of Expanded versus Non-expanded cells. **b** Samples were merged with scVI with tissue of origin as the batch key, to reduce batch effect. Left: UMAP representation and unsupervised clustering of the cells. Right: UMAP representation of the cells, coloured according to tissue origin. **c** UMAP representation of all cells from the Wu et al. cohort (Wu et al. 2021), coloured according to unsupervised clustering (left) and tissue origin (right). **d** A proliferation score (based on the expression of proliferation associated genes) of synovial membrane versus peripheral blood in the Wu et al. cohort (Wu et al. 2021). **Figure S6**. **a** Phenotypic characterization of CD8 + cells in the cohort. Proportion of the cells from ST and PB in each of the clusters shown in Fig. 2f. **b** Relative proportion of the cells from ST and PB in each cluster as identified in the UMAP in Fig. 2f, each cluster represents 100% of the cell population. **c** Left: Proportion of the cells from individual patients as identified in the UMAP in Fig. 2f. Right: Proportion of the cells from individual samples as identified in the UMAP in Fig. 2f. **d** Proportion of cells in different phases of cell cycle. **e** The expression of phenotypic markers in the clusters shown in Fig. 2f. **Figure S7. a** Clonal trafficking of CD4 + and CD8 + T cells between PB and ST. UMAP representation of all cells with intersecting clones between ST and PB as identified in the UMAP 2b. **b** UMAP representation of all cells with intersecting clones split by original patient between ST and PB as identified in the UMAP 2b. **c** Proportion of intersecting clones between ST and PB in different clusters as identified in the UMAP 2b. **d** Proportion of intersecting clones between ST and PB in different tissue of origin as identified in the UMAP 2b. **e** Antigen-specificities of the TCR repertoire from CD4 + cells matched against VDJdb. The most common target species have been highlighted. **f** Proportion of cells in different clusters identified with a unifying motif as predicted by GLIPH2. **g** Proportion of intersecting clones between ST and PB in different clusters as identified in the UMAP 2f. **h** Proportion of intersecting clones between ST and PB in different tissue of origin as identified in the UMAP 2f.

**Additional file 2. **Supplementary Table containing the following information: clinical information of the patients; identities and frequencies of CD4 + and CD8 + T cell clones intersecting between ST and PB; DE genes for Fig. 1b, 2b, and 2f; GLIPH2 predictions.

## Data Availability

The raw data used in this paper will be made available at the European Genome-phenome Archive (accession number pending). The code used to merge the scRNA-seq datasets using scVI is available in GitHub at https://github.com/DBhelsinkiuniv/HRUH-SINGLE-CELL-RA.

## Abbreviations

DE: Differentially expressed
GO: Gene ontology
IMA: Immune-mediated arthritis
JIA: Juvenile idiopathic arthritis
MNC: Mononuclear cells
PB: Peripheral blood
RA: Rheumatoid arthritis
scRNA-seq: Single-cell RNA sequencing
SM: Synovial membrane
ST: Synovial tissue
TCR: T cell receptor
Tmem: Memory T cell
Tph: Peripheral helper T cell
Treg: Regulatory T cell
UMAP: Uniform manifold approximation and projection
