# Supplementary material for: Single-cell characterisation of tissue homing CD4 + and CD8 + T cell clones in immune-mediated refractory arthritis
Source: Mol Med. 2024 Apr 9;30:48. doi: 10.1186/s10020-024-00802-1 (PMC11005137; doi:10.1186/s10020-024-00802-1)

Supplementary figure 1a

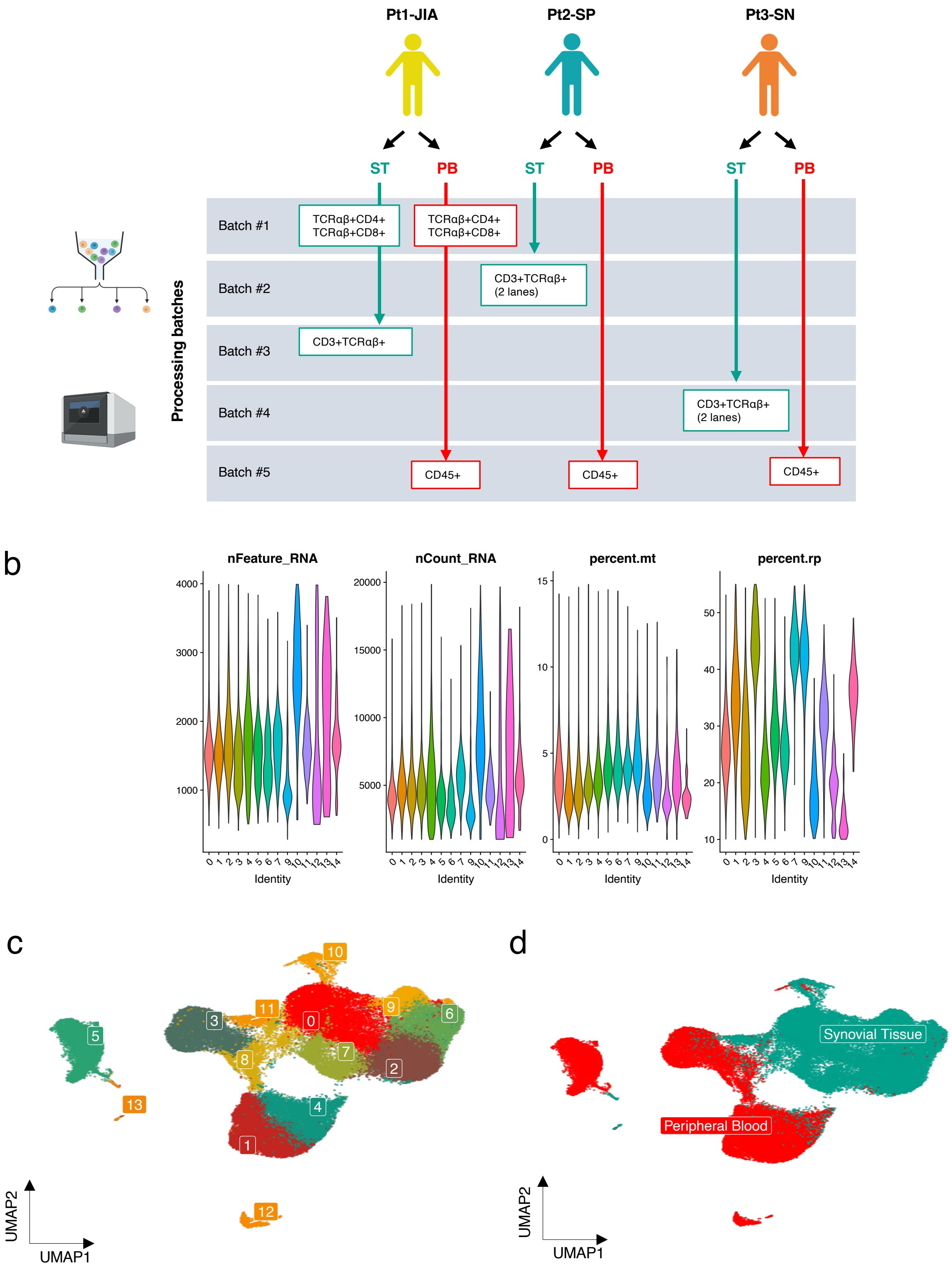

Supplementary figure 2a

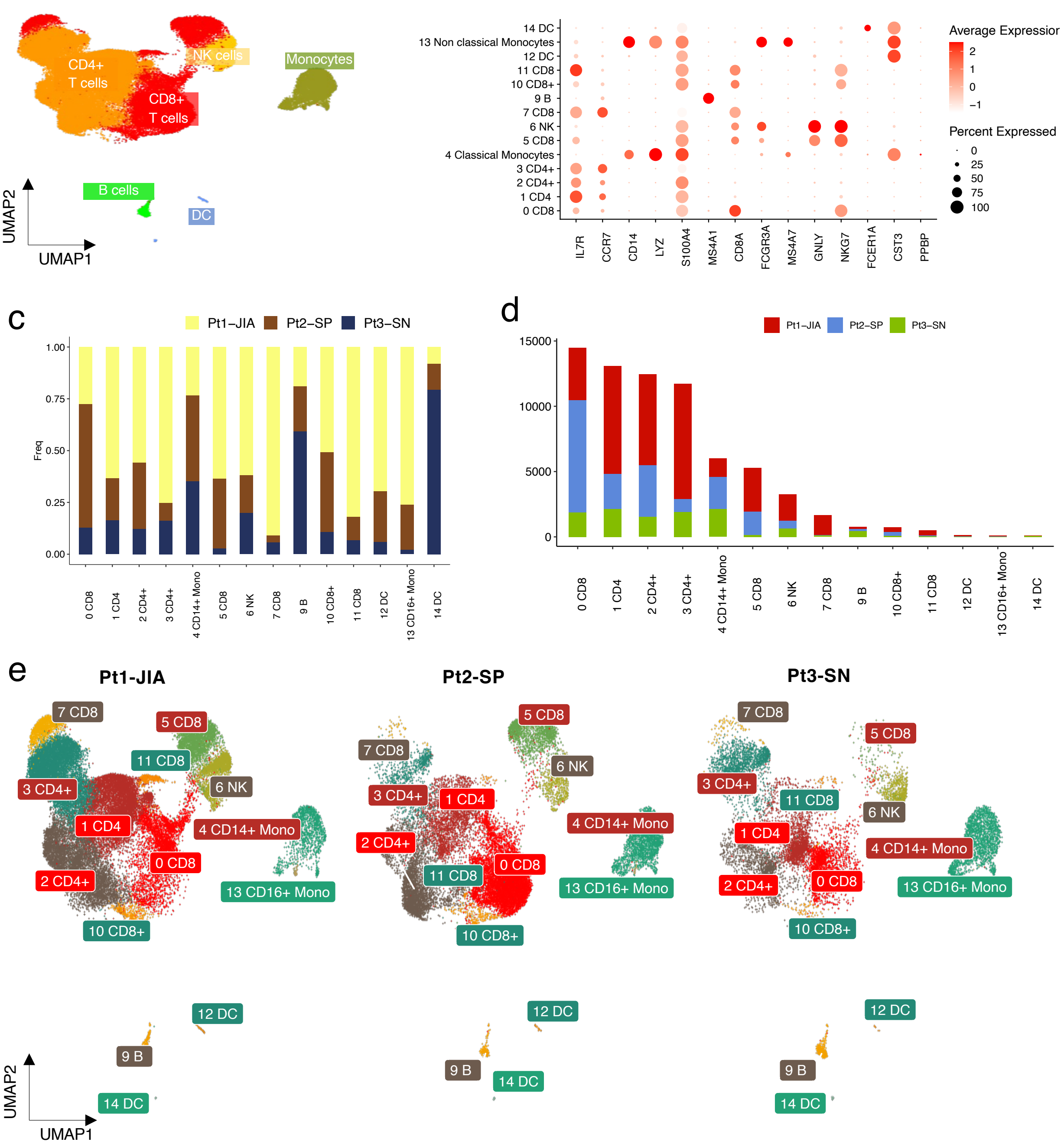

Supplementary figure 3

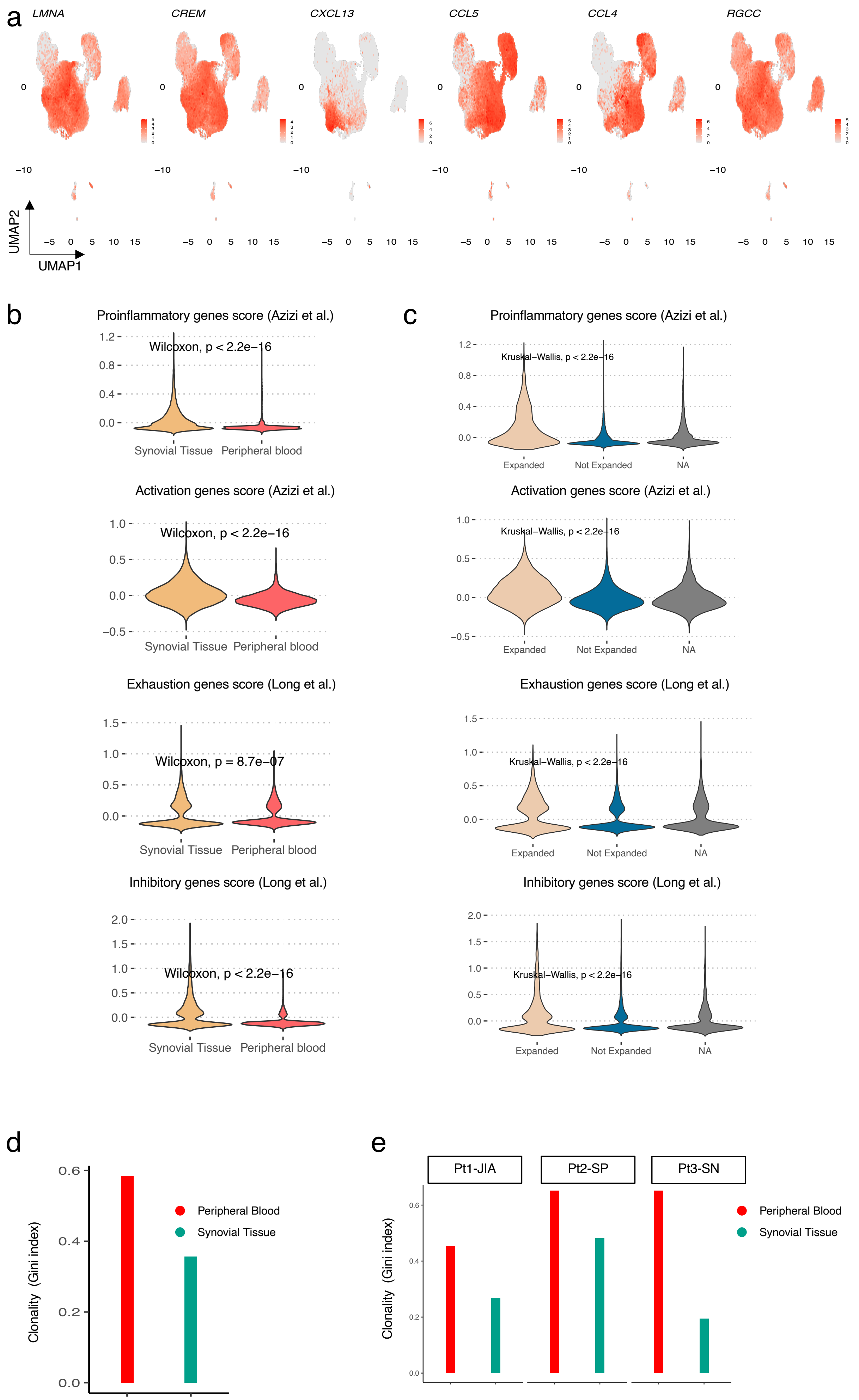

Supplementary figure 4

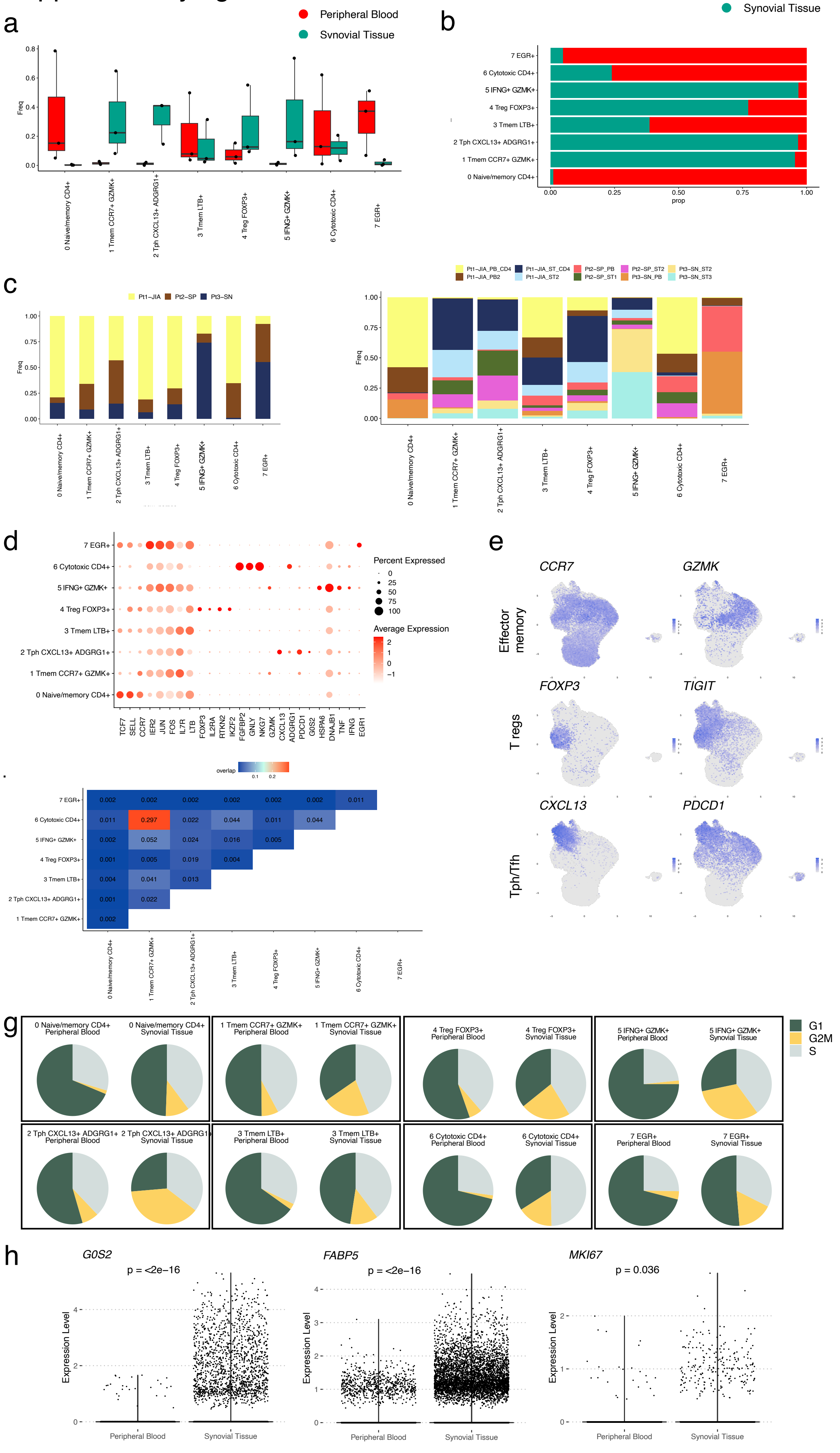

Supplementary figure 5

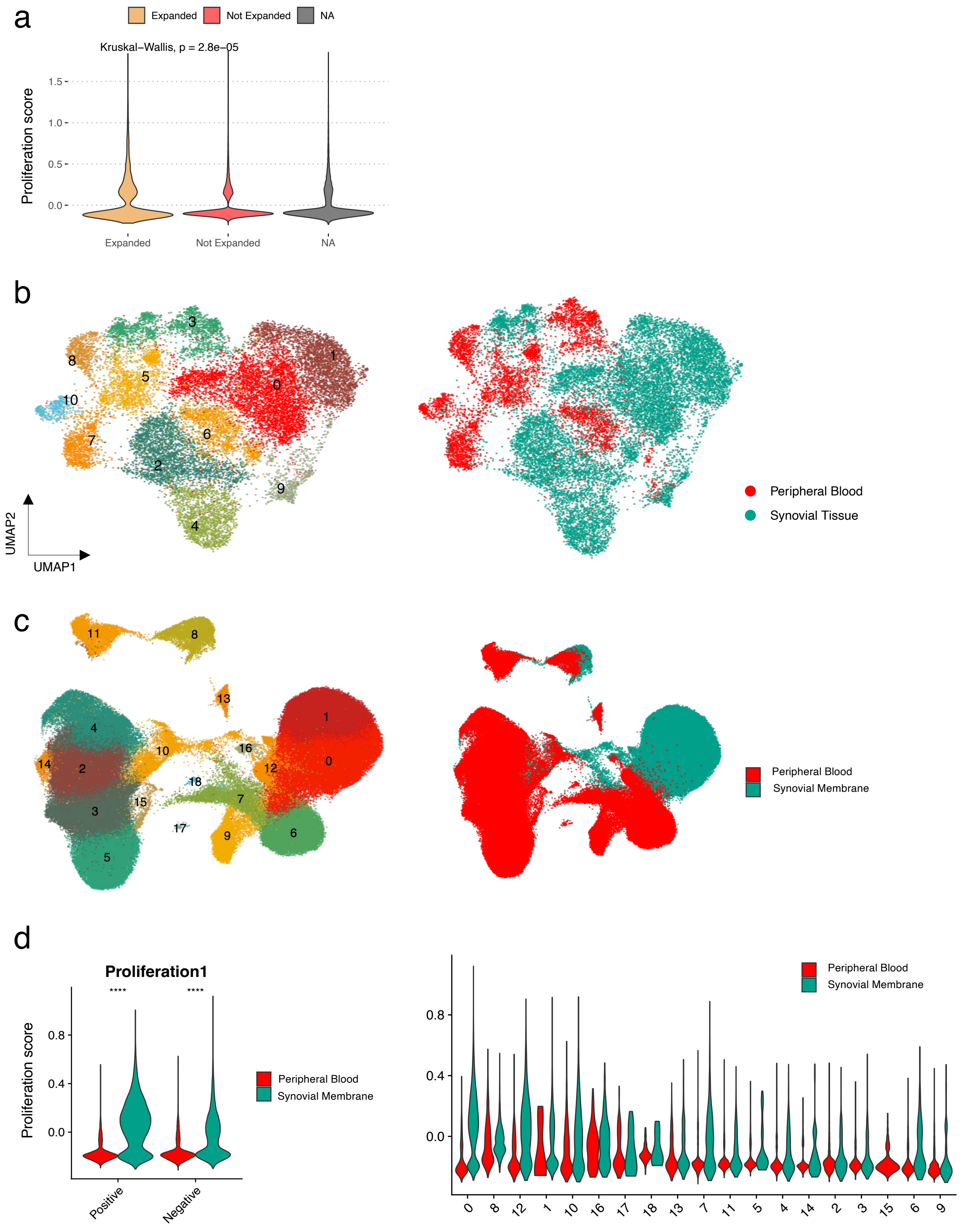

Supplementary figure 6

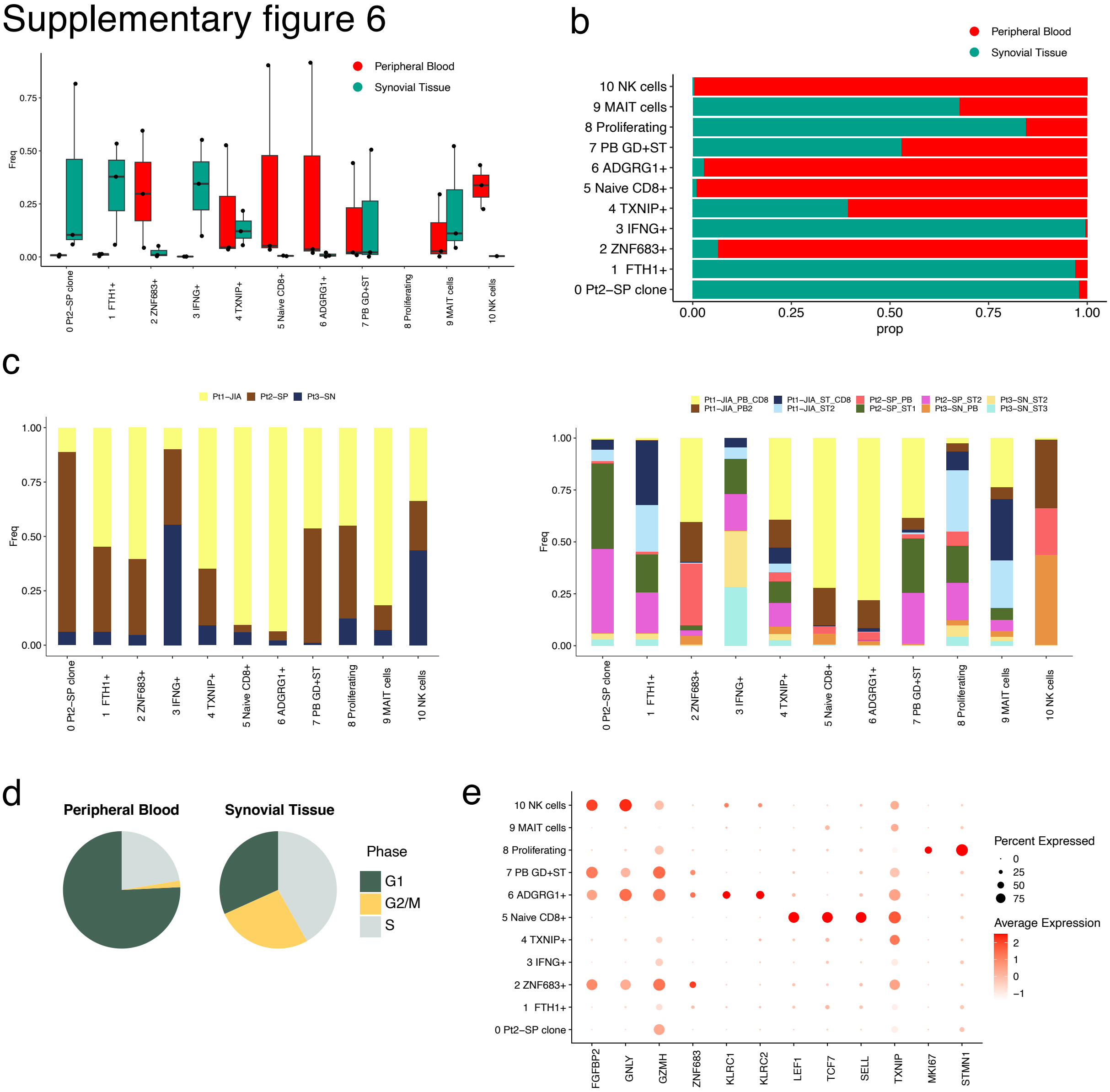

Supplementary figure 7a

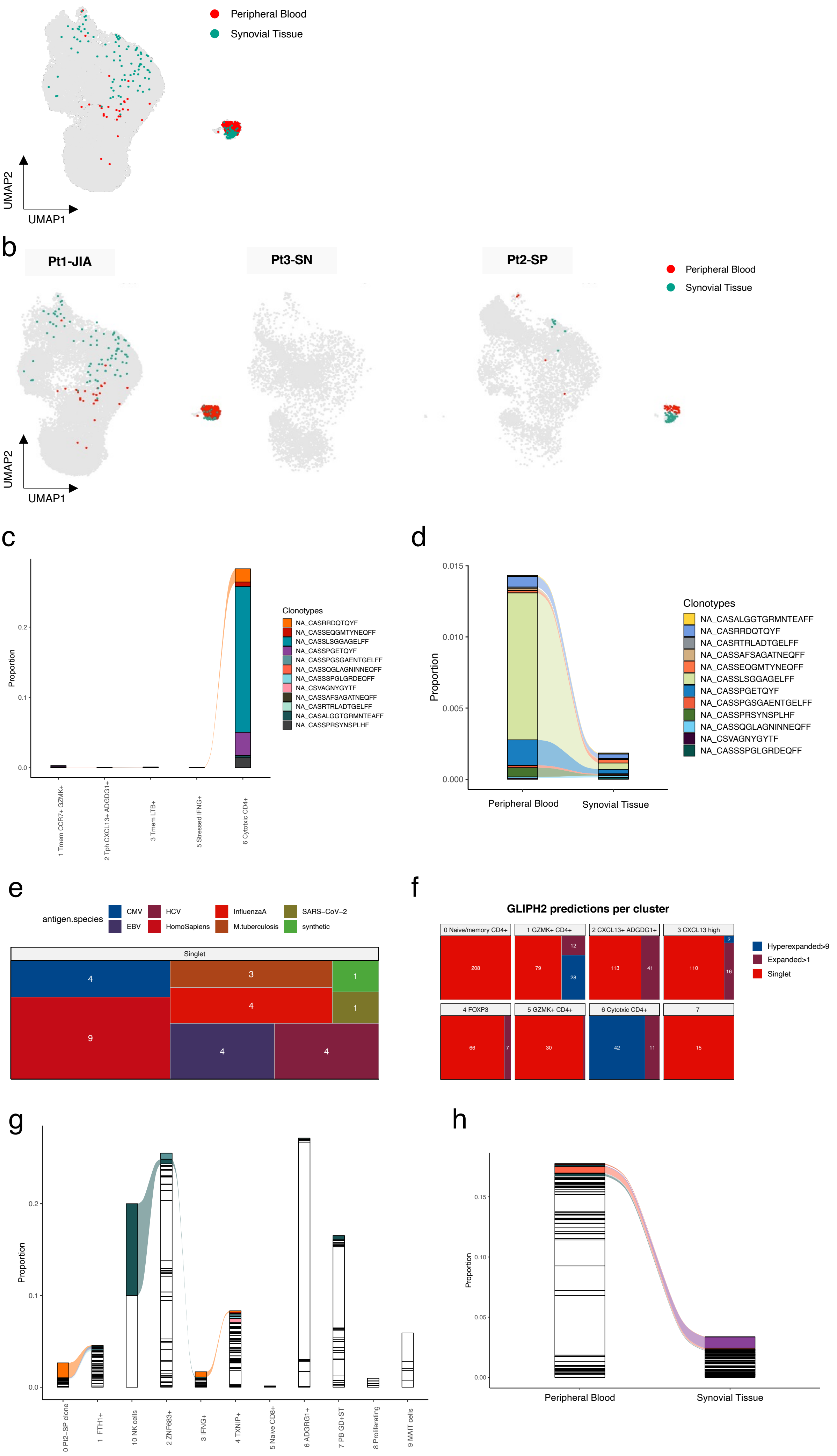

Supplement: Supplementary file 2 — Additional file 2. Supplementary Table containing the following information: clinical information of the patients; identities and frequencies of CD4 + and CD8 + T cell clones intersecting between ST and PB; DE genes for Fig. 1b, 2b, and 2f; GLIPH2 predictions. [file 10020_2024_802_MOESM2_ESM.pdf]
